# Chemoradiotherapy with or without consolidation chemotherapy using cisplatin and 5-fluorouracil in anal squamous cell carcinoma: long-term results in 31 patients

**DOI:** 10.1186/1471-2407-8-8

**Published:** 2008-01-15

**Authors:** Byoung Chul Cho, Joong Bae Ahn, Jinsil Seong, Jae Kyung Roh, Joo Hang Kim, Hyun Cheol Chung, Joo Hyuk Sohn, Nam Kyu Kim

**Affiliations:** 1 Yonsei Cancer Center, Yonsei University College of Medicine, Seoul, Korea; 2 Department of Internal Medicine, Yonsei University College of Medicine, Seoul, Korea; 3 Department of Radiation Oncology, Yonsei University College of Medicine, Seoul, Korea; 4 Department of Surgery, Yonsei University College of Medicine, Seoul, Korea

## Abstract

**Background:**

The objectives of this study were to evaluate long-term results of concurrent chemoradiotherapy (CRT) with 5-fluorouracil and cisplatin and the potential benefit of consolidation chemotherapy in patients with anal squamous cell carcinoma (ASCC).

**Methods:**

Between January 1995 and February 2006, 31 patients with ASCC were treated with CRT. Radiotherapy was administered at 45 Gy over 5 weeks, followed by a boost of 9 Gy to complete or partial responders. Chemotherapy consisted of 5-fluorouracil (750 or 1,000 mg/m^2^) daily on days 1 to 5 and days 29 to 33; and, cisplatin (75 or 100 mg/m^2^) on day 2 and day 30. Twelve patients had T3–4 disease, whereas 18 patients presented with lymphadenopathy. Twenty-one (67.7%) received consolidation chemotherapy with the same doses of 5-fluorouracil and cisplatin, repeated every 4 weeks for maximum 4 cycles.

**Results:**

Nineteen patients (90.5%) completed all four courses of consolidation chemotherapy. After CRT, 28 patients showed complete responses, while 3 showed partial responses. After a median follow-up period of 72 months, the 5-year overall, disease-free, and colostomy-free survival rates were 84.7%, 82.9% and 96.6%, demonstrating that CRT with 5-fluorouracil and cisplatin yields a good outcome in terms of survival and sphincter preservation. No differences in 5-year OS and DFS rates between patients treated with CRT alone and CRT with consolidation chemotherapy was observed.

**Conclusion:**

our study shows that CRT with 5-FU and cisplatin, with or without consolidation chemotherapy, was well tolerated and proved highly encouraging in terms of long-term survival and the preservation of anal function in ASCC. Further trials with a larger patient population are warranted in order to evaluate the potential role of consolidation chemotherapy.

## Background

Previous to work by Nigro et al. in 1974 which pioneered a concurrent chemoradiotherapy (CRT), the treatment of choice for anal squamous cell carcinoma (ASCC) was surgical resection [1]. Since then, combination chemotherapy and radiotherapy has been explored, and has led to local control rates of 60–90% and 5-year survival rates of 60–90% without the need for colostomy [2-4]. Thus, surgical resection is no longer considered a necessary treatment and has been reserved for only persistent or progressive disease.

Although increasing evidence indicates that combined modality therapy yields high local control and spares the anal sphincter, the optimal chemotherapy regimen has not been determined [5-10]. CRT with 5-fluorouracil (5-FU) and mitomicin-C (MMC) has also been evaluated in several randomized trials which demonstrated significant reductions in local failures, recurrence and subsequent colostomy [11-13]. In a study led by The United Kingdom Coordinating Committee on Cancer Research (UKCCCR), patients were randomized to either radiation to 45 Gy over 4–5 weeks or same radiation dose in combination with 5-FU and MMC [11]. Local failure rate was significantly lower in CRT arm than in the radiation alone arm. There was not, however, a statistically significant difference in overall survival. The second randomized phase III trial, conducted by the European Organization for Research and Treatment of Cancer (EORTC) Radiotherapy and Gastrointestinal Cooperative Groups, reported higher complete response rate, locoregional control rate, and colostomy-free survival rate with the addition of chemotherapy [12]. Furthermore, Intergroup trial (RTOG 87-04/ECOG1289) clearly demonstrated beneficial effects of MMC in combination regimen [13]. These studies strongly support CRT with 5-FU and MMC as the standard care for treatment of ASCC. Nevertheless, a significant proportion of patients continued to experience treatment failure and significant morbidity. In the Intergroup trial (RTOG 87-04/ECOG1289), 18% of patients experienced local-regional failure after 4 years despite treatment with a combined modality therapy [13]. Among 146 evaluable patients randomized to radiation, 5-FU, and MMC, 26% had life-threatening or lethal toxicity, including 4 fatalities. Concern regarding combination chemotherapy focused particularly on MMC, which can cause severe, life-threatening hematologic toxicity, lung toxicity, and hemolytic-uremic syndrome. Thus, there exists a need to consider alternatives to this regimen that can offer a more favorable therapeutic ratio.

Several studies suggest that cisplatin might be an effective substitute for MMC in ASCC [3,4,14-19]. A high response rate was reported in patients with locally recurrent or metastatic anal cancer treated with 5-FU and cisplatin [18]. However, reports of CRT using cisplatin-based chemotherapy remain scarce.

Consolidation chemotherapy is the prolongation of chemotherapy duration with the administration of additional drugs at the end of a defined number of initial chemotherapy cycles, after achieving a maximum tumor response in an individual patient [20]. In the absence of significant toxicity, consolidation chemotherapy is continued either for a defined time or until evidence of progressive disease. The rationale for consolidation chemotherapy is provided both by the Goldie and Coldman hypothesis [21], stating that the early use of non-cross-resistant agents might increase the probability of killing more cancer cells before resistance arises, and by the Day model [22], indicating that the most active regimens should be used as a consolidation treatment to optimize results. Recently, the usage of consolidation chemotherapy has demonstrated a potential for improving progression-free and overall survival in small cell lung cancer [23], non-small cell lung cancer [24,25], and ovarian cancer [26].

The objectives of this study were (1) to evaluate long-term results using CRT with 5-FU and cisplatin, and (2) to analyze the potential of consolidation chemotherapy for improving the outcome of patients with ASCC.

## Methods

### Patients eligibility

Between January 1995 and February 2006, a series of 31 patients presenting with ASCC were treated with CRT with 5-FU and cisplatin. Twenty-one (67.7%) of all patients received consolidation chemotherapy with the same doses of 5-FU and cisplatin, repeated every 4 weeks for maximum 4 cycles. Medical records of all patients were reviewed retrospectively.

Initial clinical work-up was performed with a careful digital examination of the anus, rectal wall, recto-vaginal septum and anoscopy or proctoscopy. All patients underwent biopsy of an anal mass or ulcer prior to referral for chemotherapy and radiotherapy. Any suspicious inguinal lymph nodes were biopsied, and pelvic CT scans and chest X-rays were performed in all patients. The staging of the tumors was done according to the UICC criteria of 2002.^27 ^The institutional review board of the hospital approved this protocol, and written informed consent was obtained from all patients.

### Treatment plan

Chemotherapy was given during the first course of radiotherapy and consisted of (1) 5-FU (1,000 mg/m^2 ^per day) on days 1 to 5, repeated on days 29 to 33 by 120-h continuous infusion (reduced to 750 mg/m^2 ^in 10 patients in whom poor tolerability to chemotherapy was anticipated by responding physicians), and (2) cisplatin (100 mg/m^2^) on days 2 and 30 (reduced to 75 mg/m^2 ^in 15 patients). Consolidation chemotherapy consisted of same regimen, repeated every 4 weeks for maximum 4 cycles. Patients were required to have an absolute neutrophil count (ANC) ≥ 1,500/μl without evidence of active infection, platelet count ≥ 100,000/μl, and resolution of any nonhematologic toxicity to less than grade 2 before receiving subsequent cycles of chemotherapy. A dose reduction of 5-FU and/or cisplatin to 10% was prescribed in cases of bone marrow suppression (absolute neutrophil count < 1000/μL and platelet count < 75,000/μL), febrile neutropenia and grade 3 or more chemotherapy-related non-hematologic toxicity. Antiemetic therapy was given routinely before the chemotherapy. Granulocyte-colony stimulating factor (G-CSF) was not planned as a prophylactic aim.

External beam radiotherapy consisted of 45 grays (Gy) in 25 fractions to the pelvis, perineum, and both inguinal lymph nodes. All patients were treated with anterior and posterior fields for the initial 45 Gy at 1.8 Gy per fraction, five times per week. The superior border was located at the bottom of the sacroiliac joints, the inferior border was at least 3 cm below the most inferior portion of the tumor or anal verge, and lateral borders covered the medial inguinal lymph nodes. A boost of 9 Gy was given to complete or partial responders.

### Evaluation of results

Tumor response was assessed by clinical examination, sigmoidoscopy, often with full-thickness biopsy, and pelvic CT scan 4 to 6 weeks following the completion of chemoradiotherapy. Response was evaluated according to WHO criteria. Complete response (CR) was defined as the complete disappearance of all measurable disease for a duration of at least 4 weeks, and partial response (PR) as a greater than 50% reduction of all measurable tumor sites. Stable disease (SD) was defined as a less than 50% reduction of tumor lesions and no progression of more than 25% in tumor diameter. The responses of initially metastatic lymph nodes were gauged by palpation by the attending physician. If palpation proved equivocal, the physician used a fine-needle biopsy at his/her discretion. In patients who showed PR at first tumor response, response evaluation was repeated with clinical examination and pelvic CT every 2 months during consolidation chemotherapy.

Patients were seen weekly during chemoradiotherapy, biweekly during consolidation chemotherapy, and thereafter every 3–6 months throughout the follow-up. Chest X-ray and pelvic CT were performed annually. Toxicities were graded according to the Common Toxicity Criteria of the National Cancer Institute, version 1.0 or 2.0 [27].

### Statistical Analysis

Local recurrence was defined as tumor recurrence in the anus. Inguinal and/or pelvic lymph nodes within the irradiated volume were also scored as local recurrence. Other sites of recurrence were scored as distant metastases. Comparison of recurrence patterns was made using a Fisher's Exact test. Disease-free survival (DFS) was defined from the date of negative biopsy to recurrence of cancer or death of any cause. Overall survival (OS) was defined from the date of diagnosis to the date of death by any cause. Colostomy-free survival (CFS) was defined as interval between the date of diagnosis and the first colostomy. Survival curves were estimated using the Kaplan-Meier method and the differences in survival between the groups were assessed by a log-rank test.

Meticulous follow-up was done to confirm that deceased patients who had been marked as having no evidence of disease (NED) did show evidence of the medical cause of death.

## Results

### Patient characteristics

Pretreatment characteristics of patients and tumors are summarized in Table 1. The median age of all patients was 57 years (range, 29–75). There were a total of 11 male and 20 female patients. The histology of all primary tumors was identified as squamous cell carcinoma arising in the anal canal. The T stages of 31 primary tumors, using AJCC criteria [28], were: two T1, seventeen T2, seven T3, and five T4 lesions. Twelve patients (38.7%) presented with synchronous inguinal node metastases, as verified by histology. Six patients with palpable perirectal nodes were presumed positive for tumor and not biopsied due to technical difficulties. About half of the patients presented with advanced cases, with 18 patients (58.1%) having tumors of 4 cm or more in maximal diameter and only 6 patients (19.4%) with tumors smaller than 4 cm without any metastatic nodes. Twenty-one (67.7%) of all patients received at least one cycle of consolidation chemotherapy.

**Table 1:** Patient characteristics.

Characteristics	Total *n *= 31
Median age (range)	57 (29–75)
Sex, *n *(%)	
Male	11 (35.5)
Female	20 (64.5)
ECOG, *n*(%)	
1	23 (74.2)
2	8 (25.8)
Tumor location	
Anal canal	31 (100)
Histologic type, *n*(%)	
Well-differentiated	3 (9.7)
Moderately-differentiated	13 (41.9)
Poorly-differentiated	6 (19.4)
Unspecified	9 (29.0)
Tumor stage, *n *(%)	
T1	2 (6.5)
T2	17 (54.8)
T3	7 (22.6)
T4	5 (16.1)
Nodal stage, *n *(%)	
N0	13 (41.9)
N1	6 (19.4)
N2	8 (25.8)
N3	4 (12.9)
Consolidation chemotherapy	4 (12.9)
Yes	21 (67.7)
No	10 (32.3)

### Treatment compliance

The median actual dose of radiation administered was 54 Gy (range, 41.4– 64.8 Gy). The anticipated duration of treatment was 42 days, whereas the actual duration was a median of 45 days (range, 31–67 days). The treatment lasted longer than 49 days in 9 patients (29%). Thus, 71% of the patients finished treatment within 7 days of the planned duration date. The primary cause of schedule delays was related to radiation dermatitis. The patient, who received a total of 64.8 Gy, had initially diagnosed as stage T4N2 and still had grossly residual tumor after CRT. Therefore, radiation oncologist decided to administer a boost of 19.8 Gy to this patient.

Ten patients received 750 mg/m^2 ^of 5-FU and 15 patients did 75 mg/m^2 ^of cisplatin instead of the initial planned dose (1,000 mg/m^2 ^of 5-FU and 100 mg/m^2 ^of cisplatin, respectively) due to advanced age (median 61 years) or poor performance status, which precluded full-dose administrations. There were 7 cases (29%) of chemotherapy delay or dose reduction due to hematologic toxicities or grade 3 urinary tract infection during chemoradiotherapy. One patient refused further chemotherapy after completion of the first cycle. Based on the initial planned dose (standard), the median actual dose intensities of 5-FU and cisplatin, administered concurrently with radiation, were 1428.6 mg/m^2^/week (range, 625.0–1666.7) and 26.7 mg/m^2^/week (range, 16.7–33.3), respectively. Median relative dose intensities (RDI) of both 5-FU and cisplatin were 0.86 (range, 0.37–1.0) and 0.80 (range, 0.50–1.0), respectively.

Twenty-one (67.7%) of all patients were treated with consolidation chemotherapy. The remaining 10 patients could not undergo chemotherapy due to poor performance status following CRT (3 patients), toxicities during CRT (2 patients with radiation dermatitis) or patients' refusal (5 patients). Of the 21 patients who started consolidation chemotherapy, 2 failed to complete all four cycles due to aggravating underlying renal insufficiency and patient's refusal, respectively. Therefore, a total of 79 cycles of consolidation chemotherapy were administered with a median of 4 cycles (range, 1–4). In this group, the median RDI of both 5-FU and cisplatin were 0.89 (range, 0.38–1.0) and 0.82 (range, 0.28–1.0), respectively. There were 8 cases (38.1%) of chemotherapy delay or dose reduction during consolidation chemotherapy. The causes of dose reduction or schedule delay were hematologic toxicity (7 patients) and asthenia (1 patient), respectively.

Overall, patient compliance was 96.8% for the CRT and 90.5% for the consolidation chemotherapy.

### Tumor response to treatments

Primary tumor responses were assessed by full-thickness biopsy in all cases. After CRT, 28 (90.3%) patients showed CR, while 3 (9.7%) showed PR. Three patients with residual disease received consolidation chemotherapy with the same regimen instead of APR. Two out of these 3 patients achieved CR after completion of consolidation chemotherapy and remained disease-free at 1 and 2 years, respectively. One patient still had metastatic disease in the inguinal lymph node as well as a residual lesion in the anal canal following consolidation chemotherapy. This patient received re-irradiation as salvage treatment, and died of progressive disease.

### Disease-free, colostomy-free, and overall survival

At a median follow-up of 72 months (range, 14–137 months), 5 recurrences and 8 deaths were recorded. Overall, 2 patients presented with a locoregional recurrence (1 in the anal canal and 1 in the inguinal lymph node). One of these patients had previously received CRT alone, while the other patient underwent 4 additional cycles of consolidation chemotherapy. The time from the end of treatment to recurrence was 12 months and 6 months for the two patients, respectively. One patient received abdominoperineal resection (APR) as a salvage therapy. However, this patient subsequently developed distant metastases and died of it. The other patient refused further treatment and is still alive after 30 months.

The two patients, who were previously treated with CRT alone and 4 cycles of consolidation chemotherapy, respectively, developed distant metastases. One had simultaneous metastases to the lung and liver 64 months after the end of treatment, and the other presented with multiple lymph node metastasis 24 months after the end of treatment. Finally, one patient who was treated with CRT alone had simultaneous local and distant metastatic disease at the time of recurrence. All three patients received platinum-based chemotherapies as salvage therapies, but died of progressive disease. The local control rate at 5 years was 87.1%. The distant control rate at 5 years was 90.3%. When we compared the recurrence pattern of patients treated with CRT alone to those treated with CRT with consolidation chemotherapy, no significant difference was found.

Five of 8 deaths were due to anal cancer. Three patients died of unrelated illness with no evidence of disease at the time of last follow-up. This was meticulously confirmed by recent follow-up physician notes, radiological reports and direct communication with family members by telephone. At the time of death, they are reported to have been disease-free for 76 months, 70 months, and 63 months, respectively.

During the follow-up period, APR was performed to resolve anal obstruction in one patient presenting with locoregional recurrence.

**Figure 1:**
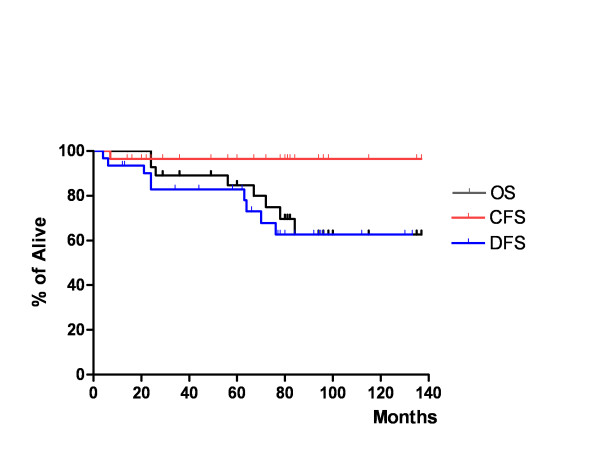
The overall, disease-free, and colostomy-free survival.

**Figure 2:**
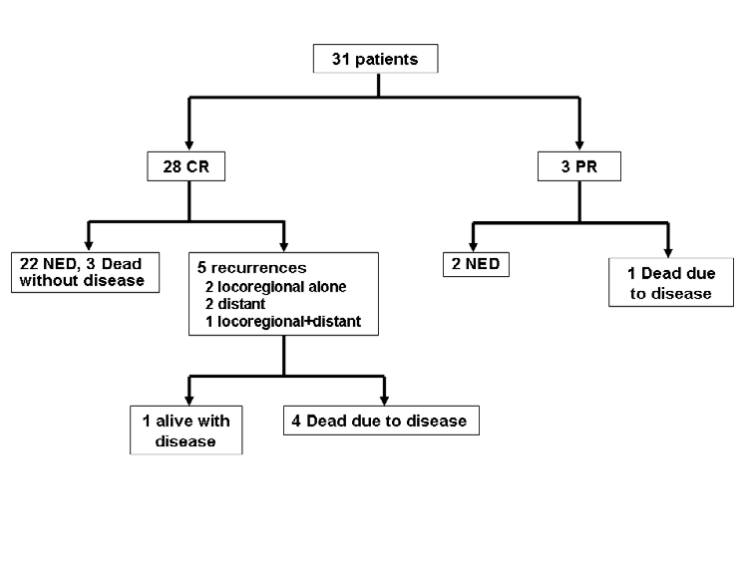
**Treatment outcome**. Abbreviation: CR, complete response; PR, partial response.

The 5-year overall, disease-free, and colostomy-free survival rates were 84.7%, 82.9% and 96.6%, respectively (Figure 1). No significant differences in 5-year OS and DFS rates between patients treated with CRT alone and CRT with consolidation chemotherapy was observed (OS 88.9% in CRT alone *vs*. 82.6%, respectively, *P *= 0.465; DFS 77.8% in CRT alone *vs*. 85.2%, respectively, *P *= 0.233). Overall treatment outcome is depicted in Figure 2.

### Toxicity

Table 2 lists the toxicities occurring during the CRT and consolidation periods.

**Table 2:** Acute toxicity evaluation per patient.

	**Chemoradiation period (n = 31)**					**Consolidation chemotherapy period (n = 21)**				
**Acute Toxicity**	**Grade (No. of patients)**				**Incidence of ≥ Grade 3**	**Grade (No. of patients)**				**Incidence of ≥ Grade 3**
	**1**	**2**	**3**	**4**	**(%)**	**1**	**2**	**3**	**4**	**(%)**

**Hematological**										
**Neutopenia**	2	3	3	0	9.7	0	3	2	5	33.3
**Anemia**	5	6	1	0	3.2	2	4	0	0	0
**Thrombocytopenia**	0	0	2	0	6.5	0	0	0	2	9.5
**Febrile neutropenia**	0	0	2	0	6.5	0	0	2	0	9.5
**Non-hematological**										
**Stomatitis**	1	4	0	0	0	3	4	0	0	0
**Diarrhea**	3	9	0	0	0	2	4	0	0	0
**Constipation**	1	1	0	0	0	0	0	0	0	0
**Nausea/vomiting**	3	16	0	0	0	3	7	0	0	0
**Asthenia**	0	2	0	0	0	0	2	1	0	4.8
**Abdominal pain**	2	4	0	0	0	0	0	0	0	0
**Infection**	0	2	1	0	3.2	0	0	0	0	0
**Dermatitis**	3	6	17	0	54.8	0	0	0	0	0

During CRT, no grade IV toxicity was observed. However, Grade III hematologic toxicities included neutropenia in 3 patients (9.7%), anemia in 1 (3.2%), and thrombocytopenia in 2 (6.5%). Febrile neutropenia occurred in 2 patients (6.5%). As expected, more than half of the patients developed grade 3 radiation dermatitis (54.8%). Other chemotherapy-related toxicities were mild to moderate and disappeared with simple medication.

In the consolidation chemotherapy group, grade III and IV hematologic toxicities were neutropenia in 7 patients (33.3%), thrombocytopenia in 2 (9.5%). Febrile neutropenia was documented in 2 patients (9.5%) and completely recovered with supportive care. There were no treatment-related mortalities.

Late toxicity was observed in 5 patients (16.1%). The most common late complications were lymphedema (3 patients, 9.7%) followed by perineal fibrosis (1, 3.2%) and deep venous thrombosis (1, 3.2%). All patients without recurrence have retained normal anal function, and no patient required a colostomy due to complications.

## Discussion

ASCC is a rare malignancy, with incidences less than 1% [29]. Therefore, the exposure of the average oncologist to ASCC is limited, even at large cancer centers. These factors explain, in part, the relative paucity of large, randomized clinical trials comparing treatment modalities for anal carcinoma.

Since the original contribution by Nigro et al [1]. in 1974, concomitant 5-FU and MMC therapy has been established as the standard in several randomized trials [11-13]. These trials have demonstrated that the inclusion of MMC resulted in superior outcomes in terms of local control and DFS rates. In a result from UKCCCR trial, local failure rate at 3 years was 39% in CRT arm compared with 61% in the radiation alone arm [11]. In the EORTC trial, CR rate was higher (80% *vs*. 54%) with the addition of chemotherapy [12]. This difference remained during follow-up, such that the 5-year estimates of locoregional control showed an 18% advantage for the combined group. Additionally, the colostomy-free survival rate in the combined group was 32% higher at 5-years. In an Intergroup trial, the addition of MMC resulted in higher CFS at 4 years (71% vs. 59%). The DFS rate at 4 years was also higher in the MMC arm than in the 5-FU alone arm (73% *vs*. 51%) [13]. However, this regimen is associated with severe toxicity in 25–50% of patients and a mortality rate of 1–3%.

Additional investigation has centered on alternate regimens, such as the substitution of cisplatin for MMC. The Cancer and Leukemia Group B (CALGB) evaluated the regimen of induction chemotherapy with 5-FU and cisplatin followed by CRT with 5-FU and MMC for patients with locally advanced anal cancer [19]. An initial report of 45 patients treated with this regimen showed a 50% 48-month CFS and DFS rate. Based on this promising data, the Radiation Therapy Oncology Group (RTOG) 98-11 trial recently evaluated the use of a cisplatin-based regimen in patients with anal cancer [30]. That trial randomized 682 patients to 5-FU and MMC plus radiation or 5-FU (1,000 mg/m^2 ^per day on days 1–4, 29–32, 57–60, and 85–88) and cisplatin (75 mg/m^2 ^on days 1, 29, 57, and 85) plus radiation (45–59 Gy beginning on day 57). There was no difference for DFS between treatment arms (HR 1.15; *P *= 0.33). OS was no different and the colostomy rate was higher in cisplatin-treated patients (HR = 1.6; *P *= 0.04), indicating that cisplatin is not superior to MMC.

Thus, this study attempts to evaluate the long-term benefit of cisplatin as a substitute to MMC in the treatment of locally advanced ASCC. Further, we investigate the potential advantage of consolidation chemotherapy as the definitive non-surgical treatment for locally advanced ASCC.

The main findings of this study indicate that CRT with 5-FU and cisplatin, with or without consolidation chemotherapy, yielded marked complete tumor response, high local control rate, and favorable long-term survival outcome. Evaluation after CRT revealed a CR rate of 90.3% and a PR rate of 9.7%. The final CR rate after consolidation chemotherapy reached 96.8%. The 5-year OS was also encouraging at 84.7%. These results compare favorably with those obtained from CRT with 5-FU and cisplatin, in which reported OS ranged from 56–73% [3,4,14-17].

Moreover, acute or late toxicities in our series seemed more favorable than those observed with 5-FU and MMC [11-13]. No grade IV toxicity or mortality was observed in our study. Furthermore, the late toxicities in our patients were much less severe than those reported from CRT with MMC. Importantly, none of our patients required a temporary or permanent colostomy for the palliation of pain or incontinence.

We deliberately did not prescribe full dose chemotherapy for the elderly or patients with poor performance status in this study. This decision was supported by reported incidence of severe complications experienced by these patients undergoing combined treatment [31,32].

Although distant disease is uncommon at presentation, 40% of deaths from anal cancer occurred with disease identified outside of the pelvis [11]. Therefore, in an effort to optimize both local and distant disease with high compliance, our center has continued to use consolidation chemotherapy with complete responders. Consolidation chemotherapy proved tolerable in our study and the majority (90.5%) of patients who started consolidation chemotherapy completed 4 cycles of consolidation chemotherapy, as scheduled. Unfortunately, we did not demonstrate any differences in survival or recurrence patterns between patients with or without consolidation chemotherapy. However, the sample size in our study was too small to statistically detect significant differences. Thus, a multi-institutional randomized trial will be required to answer questions concerning the potential role of consolidation chemotherapy in ASCC. This strategy is under evaluation in the United Kingdom, based on a similar hypothesis that such treatment can improve both local and distant control rates [33].

Historically, APR has been used for salvage attempts in the majority of patients who had either gross or microscopic residual disease following combined modality treatments [34]. However, it appears that surgical salvage is often ineffective in preventing subsequent local recurrence and death from distant metastasis [34]. A trial from Intergroup (RTOG 87-04/ECOG1289) [13] reported that 27 patients with residual disease after CRT were successfully treated with salvage CRT using 5-FU and cisplatin. Of 24 assessable patients, 12 attained CR and five of the 12 did not require APR at 3 years. This study also demonstrates a possible benefit of salvage chemotherapy using 5-FU and cisplatin in patients with residual disease following initial CRT. In our study, additional chemotherapy was able to salvage, without colostomy, 2 of 3 patients who failed to attain CR after initial CRT, demonstrating the chemosensitive nature of ASCC [35,36].

## Conclusion

Our study shows that CRT with 5-FU and cisplatin, with or without consolidation chemotherapy, was well tolerated and proved highly encouraging in terms of long-term survival and the preservation of anal function in ASCC. These data further support the indication to treat anal cancer with a combination of 5-FU and cisplatin as primary treatment. However, the activity of this regimen needs to be tested in phase III randomized trials before it can be implemented in standard clinical practice. The second UK phase III anal cancer trial (ACT II) are currently in progress for that purpose [37].

## Competing interests

The author(s) declare that they have no competing interests.

## Authors' contributions

BCC and JBA, JS, JKR, JHK, HCC, JHS, and NKK have made substantial contributions to conception and design of the study. BCC, JBA, WNS, JS, JKR, JHK, HCC, JHS, NKK carried out acquisition of data. BCC, WNS, and JBA carried out analysis and interpretation of data. BCC and JBA have been involved in drafting the manuscript. BCC, JBA, WNS, JS, JKR, JHK, HCC, JHS, NKK have given final approval of the version to be published.

## Pre-publication history

The pre-publication history for this paper can be accessed here:


